# Heterozygous Mutations in Aromatic Amino Acid Synthesis Genes Trigger TOR Pathway Activation in *Saccharomyces cerevisiae.*

**DOI:** 10.17912/micropub.biology.000685

**Published:** 2022-11-16

**Authors:** Makailyn G Schoonover, Eon C Chilson, Erin D Strome

**Affiliations:** 1 Northern Kentucky University

## Abstract

The highly conserved complexes of Target of Rapamycin (TORC1 and TORC2) are central regulators to many vital cellular processes including growth and autophagy in response to nutrient availability. Previous research has extensively elucidated exogenous nutrient control on TORC1/TORC2; however, little is known about the potential alteration of nutrient pools from mutations in biosynthesis pathways and their impact on Tor pathway activity. Here, we analyze the impacts of heterozygous mutations in aromatic amino acid biosynthesis genes on TOR signaling via differential expression of genes downstream of TORC1 and autophagy induction for TORC1 and TORC2 activity.

**Figure 1. Analysis of heterozygous mutations on Tor pathway signaling:**
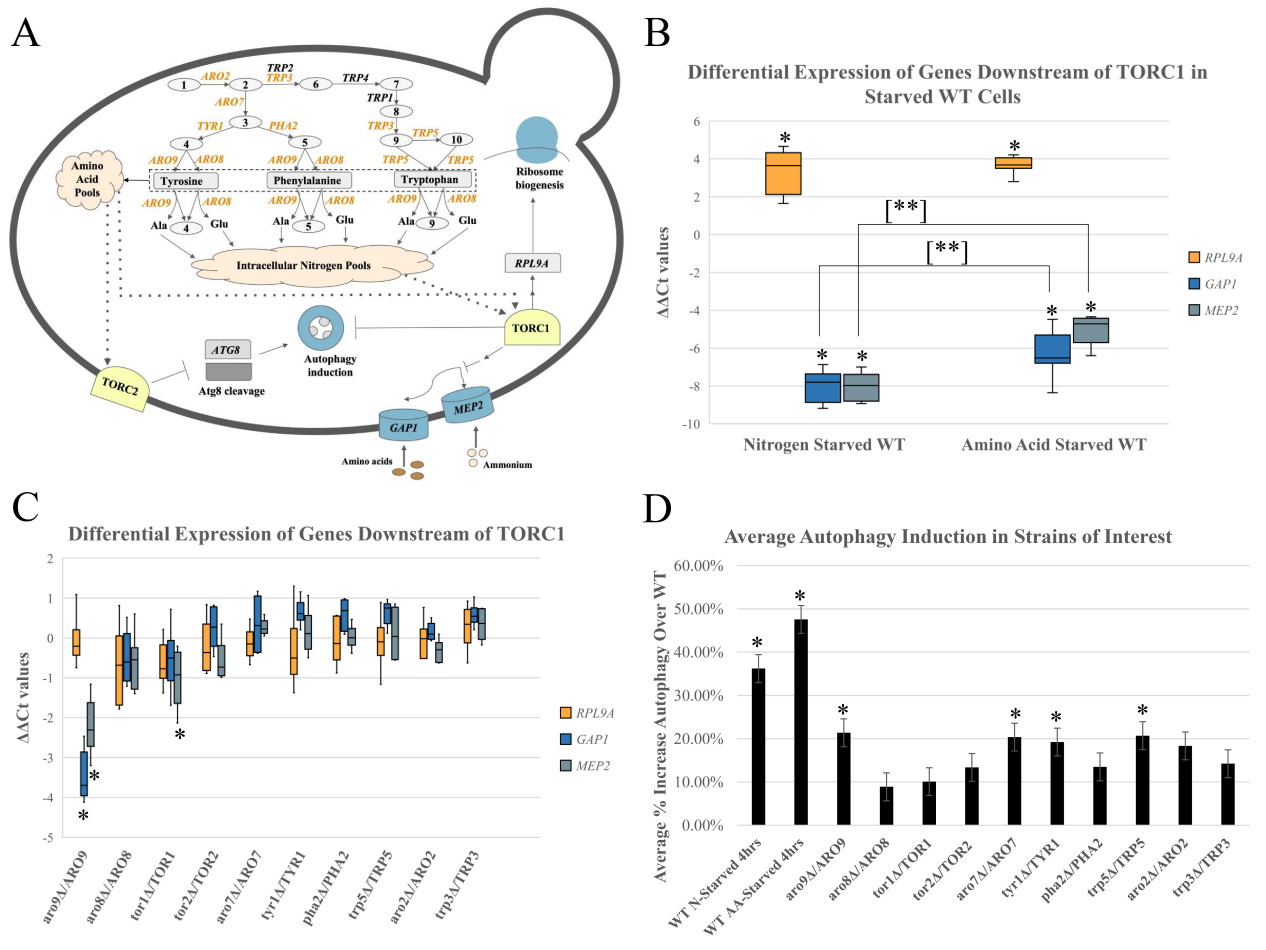
A. Aromatic amino acid biosynthesis pathway and Tor pathway signaling. Strains with heterozygous mutations in the genes listed in orange were created for the following experiments. Intermediates listed as numbers in the figure are as follows: 1. 5-o-1-carboxyvinyl-3-phosphoshikimate 2. Chorismate 3. Prephenate 4. P-Hydroxyphenyl Pyruvic Acid 5. Phenylpyruvate 6. Anthranilate 7. N-5-Phospho-B-d-ribosylanthranilate 8. 1-(2-Carboxylphenyl amino)-1-deoxy-1-ribulose-5-phosphate 9. 3-Indoyl-glycerol phosphate 10. Indole B. ΔΔCt values from qRT-PCR trials for nitrogen starved and amino acid starved wildtype strains, depicting differences in gene expression for *RPL9A* , *GAP1* , *MEP2* . Positive values indicate decreased expression and negative values indicate increased expression compared to wildtype. In each box plot, the bottom whisker shows the minimum value, the bottom of the box represents quartile 1 (Q1), the middle of the box is the median (Q2), the top of the box represents the upper quartile (Q3), and the top whisker shows the maximum value. The single asterisk indicates a statistically significant difference from wildtype, and the double asterisk indicates a statistically significant difference between the bracket connected strains. C. ΔΔCt values of qRT-PCR trials for mutant strains of interest. Formatted identical to Fig. 1B. D. Percent increase in autophagy induction compared to wildtype from western blot analysis. Asterisks signify statistically significant results compared to wildtype.

## Description

The Tor pathways, centrally regulated by Target Of Rapamycin Complexes, TORC1 and TORC2, have been heavily characterized in their distinct and significant involvement in many cellular pathways regulating cell growth and maintenance in response to various stimuli. *Saccharomyces cerevisiae* has two TOR genes, *TOR1* and *TOR2* , and their products, the Tor1 and Tor2 proteins, respectively, function redundantly in the formation of TORC1, while TORC2 is formed from the incorporation of Tor2 protein only (Heitman et al. 1991; Loewith et al. 2002; Wedaman et al. 2003). Mechanistic studies of the anti-cancer drug, rapamycin, helped identify the TOR genes and found that rapamycin binds to and renders TORC1, and not TORC2 inactive, initiating a myriad of studies that have used rapamycin to examine TORC1 function and distinguish it from TORC2 (Heitman et al. 1991; Stan et al. 1994). The Tor pathways are highly conserved and crucial in maintaining cellular homeostasis, as aberrant functioning of these pathways is present in many pathological conditions in humans, making TORC1 and TORC2 a valuable topic of study as important cell regulators (Tatebe and Shiozaki 2017).

Function, regulation, and downstream effectors of TORC1 and TORC2

TORC1 controls temporal aspects of cellular growth processes including G2/M cell cycle progression, transcriptional regulation of nutrient responsive genes, translation, ribosome biogenesis, protein synthesis and degradation, nutrient transport, and negative regulation of autophagy response (Barbet et al. 1996; Noda and Ohsumi 1998; 1998; Beck and Hall 1999; Powers and Walter 1999; Nakashima et al. 2008; Hatakeyama et al. 2019). TORC2 controls spatial aspects of cellular growth including actin polarization, endocytosis, negative regulation of calcineurin activity, and positive regulation of autophagy during amino acid starvation (deHart et al. 2003; Kamada et al. 2005; Vlahakis and Powers 2014; Rispal et al. 2015; Riggi et al. 2019: 2). There is extensive evidence showing a conserved characteristic of direct regulation of the Tor pathway by nutrient availability, including disruption of TORC1 mimicking the effects of nutrient starvation (Beck and Hall 1999; Rohde et al. 2008; Wang and Proud 2009; Conrad et al. 2014; McDaniel et al. 2017). TORC1 and TORC2 have distinct localizations, with TORC1 at vacuolar membranes and TORC2 at the plasma membrane, potentially due to the distinct functions and regulations of the complexes (Reinke et al. 2004; Aronova et al. 2007; Sturgill et al. 2008). The vacuole in yeast is a major nutrient reservoir, and a primary site for amino acid storage (Russnak et al. 2001; Sekito et al. 2008). TORC1 is known to be regulated by amino acids through direct interaction with the vacuolar membrane localized EGO complex (that plays a role in nitrogen sensing), and nitrogen through a less understood mechanism via the Nitrogen Catabolite Repression (NCR) pathway in response to the quality of nitrogen sources and a potential feedback loop by TORC1 downstream effectors, Npr1p and Par32p, for quantity of nitrogen sources (Binda et al. 2009; Jeong et al. 2012; Sekiguchi et al. 2014; Li et al. 2017). Regulation of TORC2 has been less characterized. Recent studies have proposed that TORC2 activity is not as tightly regulated by nutrients as TORC1, however, it has been shown that TORC2 function is specifically sensitive to amino acid, and not nitrogen, levels (Ecker et al. 2010; Vlahakis and Powers 2014; Vlahakis et al. 2016). Located downstream of, and negatively regulated by TORC1, the NCR pathway senses quality of nitrogen sources and appropriately adjusts metabolic function to optimize fitness (Reviewed in Magasanik and Kaiser 2002). In the presence of preferred nitrogen sources, the NCR pathway increases expression of genes involved in preferred nitrogen source uptake and metabolism and represses genes involved in non-preferential nitrogen sources, and vice versa under poor nitrogen sources (Coffman et al. 1997; Cooper 2002). High affinity permeases, *GAP1* and *MEP2* are transcriptionally controlled downstream of TORC1, through the NCR pathway (Cooper 2002; Airoldi et al. 2016). Gap1p, located in the plasma membrane and primarily present under conditions of poor nitrogen sources, acts as an general amino acid transporter and also functions as a nutrient senser for the PKA pathway (Donaton et al. 2003; Risinger et al. 2006). Gap1p has also been demonstrated to be one of the main uptake systems for aromatic amino acids (Iraqui et al. 1999). Mep2p, like Gap1p, localizes to the plasma membrane and is primarily present in the presence of poor nitrogen sources due to transcriptional regulation through the NCR pathway. Mep2p is a conserved ammonium permease involved in the regulation of pseudohyphal growth in response to nitrogen starvation, and similar to Gap1p, has been shown acts as a nutrient sensor for the PKA pathway (Marini et al. 1997; Lorenz and Heitman 1998; Van Nuland et al. 2006).

TORC1 and TORC2 regulation of autophagy

Both TORC1 and TORC2 have roles in autophagy regulation, however, they regulate it through distinct pathways and are stimulated under different conditions (Vlahakis and Powers 2014). Under normal cellular conditions, autophagy occurs at a basal rate and is essential in promoting longevity and maintaining cellular homeostasis. Moreover, in conditions of cellular stress, e.g. starvation, autophagy activity is increased and crucial to modulate macromolecule degradation and recycling to establish viable conditions (Das et al. 2012). Impaired autophagy regulation has significant cellular impacts, as it has been implicated in many pathological conditions, and therefore remains an important focal point in research (Mizushima and Levine 2020). Cells with faulty autophagy are unable to maintain necessary amino acid levels in stress-induced conditions, and fail to conduct protein synthesis, resulting in serious cellular consequences (Onodera and Ohsumi 2005). Under normal conditions, i.e., no cellular stress, TORC1 is active and inhibits autophagy through phosphorylation of Atg13p, a subunit of the autophagosome (Cebollero and Reggiori 2009; Kabeya et al. 2009; Kamada et al. 2010). Although less characterized, TORC2 has implicated roles in preventing autophagy induction in nutrient replete conditions through its downstream effector Ypk1p limiting long-chain base phosphate levels by promoting synthesis of ceramides (Zimmermann et al. 2013; Muir 2015). In conditions of cellular stress, e.g. starvation, autophagy activity is increased and crucial to modulate macromolecule degradation and recycling to establish viable conditions (Das et al. 2012). Autophagy is activated when the TORC1 pathway is stimulated by nitrogen starvation, and/or when the TORC2 pathway is stimulated by amino acid starvation, especially auxotrophic amino acid starvation (Vlahakis and Powers 2014). This distinct regulation provides cells with a tailored response to varying nutrient conditions to promote cell survival. Previous research studying the impacts of nutrients on Tor signaling have used nutrient-limited media, not mutations in amino acid biosynthesis genes, specifically heterozygous mutations and those involved in aromatic amino acids. Our work seeks to characterize heterozygous mutations in aromatic amino acid biosynthesis genes for their impact on Tor signaling pathways and determine whether they might cause effects sensed through TORC1 and/or TORC2.

Heterozygous mutation of *ARO9* alters TORC1-dependent gene expression

We hypothesized that heterozygous mutations in aromatic amino acid biosynthesis genes could decrease nutrient availability to a point that triggers Tor pathway activation. We first sought to test for changes in genes downstream of TORC1, and used qRT-PCR to examine changes in expression levels of TORC1-dependent genes: *RPL9A* , *GAP1,* and *MEP2* . These genes were ideal to measure TORC1 activity because of their known transcriptional regulation by TORC1 and shown sensitivity to nutrient levels fed through TORC1. First, to establish the sensitivity of these assays in our yeast strains, we analyzed our wildtype (WT) strain alone, and after starvation from either nitrogen or amino acids for 2 hours, during log phase growth, to determine the extent to which expression of these genes is altered. Starvation treatments (Fig. 1B) revealed the extent of measurable changes in these genes, consistent with known TORC1 regulation, as the ribosomal subunit gene *RPL9A* shows significant decreases in its expression on the order of 10-20 fold, and the nutrient permeases *GAP1* and *MEP2* can be measured for increases in their expression on the order of 150-250 fold. To analyze the significance of the data, two-tailed t-tests with unequal variance were performed with a Benjamini-Hochberg correction set to a 5% false discovery rate. Significant differences are indicated with asterisks in Fig. 1B and C. Expression of *MEP2* and *GAP1* were both found to be significantly increased in the amino acid starved WT, nitrogen starved WT, and the *aro9∆/ARO9* mutant cells. *RPL9A* expression change was only found significant in amino acid starved WT and nitrogen starved WT cells. As seen in Fig. 1B, the effect of nitrogen starvation on WT produced more profound gene expression changes than amino acid starvation, including a statistically significant difference in *GAP1* and *MEP2* expression. We would expect both conditions to produce significant expression changes due to TORC1’s known regulation by amino acids and nitrogen. However, it is interesting to note the greater impact under the nitrogen starvation condition, potentially due to nitrogen sources having a greater effect on TORC1 activity. It would also explain the result difference we see between *aro9∆/ARO9* and other strains due to their differing functions leading to different nutrient sources. The results of *aro9∆/ARO9* compared to the heterozygous loss of other aromatic amino acid biosynthesis genes (Fig. 1C) is potentially explained by the distinct catabolic role and regulation of *ARO9* . The enzymes produced from *ARO8* and *ARO9* are involved in reversible reactions, performing both anabolic and catabolic functions. They are aromatic aminotransferase isozymes; they have similar roles but are distinctly regulated, use different amino donors, and have different Km values for the aromatic amino acids. *ARO8* Km value for tyrosine and phenylalanine is 0.3, whereas, for tryptophan, it is 6 (Kradolfer et al. 1982). The Km for *ARO9* is similar for all three aromatic amino acids, falling around 0.2-0.4 (Kradolfer et al. 1982). Expression of *ARO8* is constitutive and controlled by general amino acid control, whereas *ARO9* is inducible and regulated by aromatic amino acid levels and quality of nitrogen sources through the NCR pathway (Iraqui et al. 1998). *ARO9* is transcriptionally induced under conditions of poor nitrogen sources, such as proline or urea, to retrieve nitrogen from amino acids (Iraqui et al. 1999; Staschke et al. 2010). It has also been demonstrated that in strains with an *aro8∆/ARO8* heterozygous mutation, *ARO9* is able to maintain phenylalanine and tryptophan levels to support normal growth (Urrestarazu et al. 1998). This inducible redundancy in *ARO9* function for *ARO8* would help to explain why our results were significant for *aro9∆/ARO9* and not *aro8∆/ARO8* . Although similar in function, their distinct regulation mechanisms and properties indicate a tailored utilization of these enzymes to meet specific cellular needs; and their possession of different capacities for haploinsufficiency effects. It has been postulated that the product of *ARO9* is primarily involved in tryptophan catabolism, but plays important roles in the catabolism of all three aromatic amino acids (Iraqui et al. 1999). The product of *ARO9* , aromatic aminotransferase II, catalyzes the first step of aromatic amino acid catabolism (Kradolfer et al. 1982; Iraqui et al. 1999). Aromatic aminotransferase II exerts its transaminase function on aromatic amino acids, supplying glutamate, a preferred nitrogen source, among other nitrogen sources, for the cell as a byproduct (Hazelwood et al. 2008; Crépin et al. 2012). Heterozygous deletion of *aro9∆/ARO9* could therefore limit the transaminase function, thereby limiting the preferred nitrogen source available in cells. As previous research has shown the tight regulatory role nitrogen has on TORC1, this depletion of preferred nitrogen would presumably limit TORC1 functioning. Downstream of and negatively regulated by TORC1, the NCR pathway responds to quality of nitrogen sources through four GATA-type transcription factors; activators Gln3p and Gat1p, and repressors Dal80p and Gzf3p, mediated through transcriptional control and localization by Ure2p (Hofman-Bang 1999; Georis, Feller, Vierendeels, et al. 2009). In the case of poor nitrogen sources, TORC1 phosphorylation of Gln3p is suppressed, allowing its nuclear localization, leading to the upregulation of *GAP1* and *MEP2* (Bertram et al. 2002; Georis, Feller, Tate, et al. 2009). Therefore, the decreased aromatic aminotransferase II present in the *aro9∆/ARO9* strain leads to decreased TORC1 activity as measured through the decreased expression of *GAP1* and *MEP2* , demonstrating results consistent with previous research and emphasizing the influence Aro9p has on TORC1. As the qRT-PCR analysis is only measuring genes downstream of TORC1, the lack of significance for *RPL9A* and *GAP1* expression change in *tor1∆/TOR1* and *tor2∆/TOR2* strains can be explained by the redundancy of Tor1p and Tor2p in the formation and function of TORC1. Interestingly, *tor1∆/TOR1* , and not *tor2∆/TOR2* showed significant expression change for *MEP2* . Although *GAP1* and *MEP2* share similar transcriptional regulation, previous studies have reported higher levels of fold change for *MEP2* compared to *GAP1* under rapamycin treatment and nitrogen starvation, indicating an elevated sensitivity to changes in TORC1 signaling for *MEP2* (Cardenas et al. 1999; Matsui et al. 2013). Although Tor1p and Tor2p are redundant in the formation of TORC1, there are reports of Tor1p performing distinct functions from Tor2p, including the dependency of *TOR1* expression, and not *TOR2* , for viability in strains mutated for protein sorting gene *PEP3* (Zurita-Martinez et al. 2007). Although the mechanisms are not known, this indicates a potential for additional and distinct regulation of *MEP2* through TORC1 via Tor1p specific action. Documented expression differences in *MEP2* and *GAP1* combined with this potential regulation by Tor1p on *MEP2* could help explain why *tor1∆/TOR1* showed significant expression changes for *MEP2* and not *GAP1* . The lack of statistical significance for *tor2∆/TOR2* on *MEP2* expression points to *TOR2* being less prone to haploinsufficiency effects and supports the idea of varying roles of Tor1p and Tor2p in TORC1 functioning.

Heterozygous mutation in aromatic amino acid biosynthesis and TOR pathways induce the autophagy response

Due to both TORC1 and TORC2’s known functions in autophagy regulation, we were interested to see if heterozygous mutations in aromatic amino acid biosynthesis genes could lead to increased autophagy induction. To determine if heterozygous mutations in our genes of interest result in changes able to impact autophagy induction, we utilized western blot analysis to measure the processing of a GFP-Atg8 fusion protein. In this system, induction of autophagy results in cleavage of the GFP-Atg8 protein to GFP and Atg8p, where Atg8p is then activated allowing it to participate in the formation of the autophagosome (Weidberg et al. 2010; Matsui et al. 2013). From these analyses with two-tailed independent t-tests with unequal variance and Benjamini-Hochberg correction, we determined that the wildtype samples starved for nutrients for prolonged periods (4+ hours) elicited the greatest increases in autophagy induction, consistent with previous studies (Ecker et al. 2010; Matsui et al. 2013). In comparison, strains mutated for either of the TOR genes or biosynthesis genes of the aromatic amino acid pathway had varying impacts on autophagy induction. Strains harboring heterozygous mutations for biosynthetic genes at various points in the pathway, specifically for genes *ARO7, TYR1, TRP5,* and *ARO9* , induced significant increases in the autophagy response, while mutations at other points in the pathway failed to elicit a response. Results are shown in Fig. 1D. The increase in autophagy induction within our starved wildtype strains is as we expected, however, it is interesting to note the greater percent increase in amino acid starved cells compared to nitrogen starved cells. As previous work has distinguished autophagy induction from TORC1 and TORC2 based on nutrient conditions, specifically that the TORC1 pathway to autophagy is stimulated by nitrogen starvation, and the TORC2 pathway is stimulated by amino acid starvation, (especially auxotrophic), these results support this finding of differing regulation (Vlahakis and Powers 2014; Yu et al. 2015; Noda 2017). Our *tor2∆/TOR2* and *tor1∆/TOR1* strains did not produce a statistically significant autophagy induction. This is not concerning for two reasons. First, the redundancy of Tor1p and Tor2p in the formation of TORC1 and second, the extent of the *TOR2* heterozygous mutation may have a slight impact on TORC2 functionality but not enough to elicit a statistically significant increase in autophagy induction under nutrient sufficient conditions. Based on qRT-PCR data collected for *aro9∆/ARO9* , showing a significant impact on TORC1 activity, we hypothesize the increased autophagy induction is at least partly due to effects downstream of TORC1. As heterozygous mutation of *ARO9* causes decreased TORC1 activity, the phosphorylation of Atg13p by TORC1 is suppressed, allowing for the formation of the autophagosome and autophagy induction thereafter. However, because Aro9p also has anabolic functions in the formation of aromatic amino acids, autophagy induction through TORC2 cannot be ruled out. We hypothesize our remaining mutants with significant increases in autophagy: *aro7∆/ARO7, tyr1∆/TYR1,* and *trp5∆/TRP5* , occur through the downstream effects of TORC2 signaling. Both because we did not see them able to induce a significant reduction in TORC1 activity through control on *RPL9A, GAP1,* or *MEP2* and because of their roles in amino acid production. The products of these genes all act either at the last step to produce a specific amino acid or in a differentiation step to produce a specific amino acid, or group, over another. Individual deletions of *TRP5* and *TYR1* in haploid cells have been shown to create auxotrophic cells for tryptophan and tyrosine, respectively, indicating their necessity for aromatic amino acid production (Miozzari et al. 1978; Hassing et al. 2019). With heterozygous mutations in these genes, we hypothesize the decreased pools of the respective aromatic amino acids decrease TORC2 stimulation, leading to the observed increase in autophagy induction. As *ARO7* functions at a branch point, leading to tyrosine and phenylalanine production, a mutation in *ARO7* could decrease pools of tyrosine and phenylalanine available, again decreasing amino acids available for TORC2 stimulation. This distinct regulation would presumably provide cells with a tailored response to varying nutrient conditions to promote cell survival.

Conclusion

Due to the central and highly conserved roles the Tor pathways regulate, and the detrimental effects observed following faulty functioning of these pathways, continued research on this key regulatory pathway is warranted. Previous research studying the impacts of nutrients on Tor signaling have used nutrient-limited media, not mutations in amino acid biosynthetic genes, specifically those involved in aromatic amino acids. A previous study identified *ARO1* , among others, as an underlying allelic variant involved in differences in nitrogen consumption between strains (Cubillos et al. 2017) Aro1p is involved in the biosynthesis of chorismite, a precursor to synthesis of aromatic amino acids, phenylalanine, tryptophan, and tyrosine. As Tor signaling is regulated by nutrients, namely nitrogen and amino acids, this is interesting as it shows the connection between synthesis and implicated utilization of aromatic amino acids in the Tor pathway in *S. cerevisiae* . Additionally, aromatic amino acids have been associated with Tor signaling through Gcn4p, a transcriptional activator of aromatic amino acid biosynthetic genes and downstream effector of TORC2, that acts through the GAAC (Braus 1991; Zaborske et al. 2010). Furthermore, homozygous mutations of *ARO1* result in a block of sporulation, relieved by addition of aromatic amino acids to the medium (Lucchini et al. 1978). Studies have shown sporulation requires autophagy, as ATG knockouts do not sporulate (Tsukada and Ohsumi 1993; Thumm et al. 1994; Barve and Manjithaya 2021). This work established a connection, between aromatic amino acids and the autophagy branch of Tor pathway signaling. Our research using *S. cerevisiae* diploid strains assessing impacts on Tor signaling from heterozygous mutations in aromatic amino acid biosynthesis genes expands this understanding of the Tor pathways’ regulations and downstream effects. Overall, we found that heterozygous mutation of *ARO9* led to significant perturbation of TORC1 signaling through gene expression changes downstream of TORC1. We hypothesize the distinct significance of our *aro9∆/ARO9* strain is due to the anabolic function of *ARO9* , providing both aromatic amino acids and nitrogen sources for the cell. As previous research has shown TORC1 regulation by both amino acids and nitrogen sources, this work helps specify TORC1 nutritional regulation, as we found nitrogen starved WT had showed a greater alteration to TORC1 downstream effectors compared to amino acid starved WT. These findings highlight the tight regulation between Aro9p and TORC1, and nutritional regulation of TORC1. Additionally, we found that heterozygous mutations in *ARO9, ARO7, TYR1,* and *TRP5* resulted in statistically significant increases in autophagy induction. We hypothesize that the *aro9∆/ARO9* strain led to increased autophagy induction at least partly through TORC1 supported by our qRT-PCR data results, while the remaining mutants more likely acted through TORC2. *ARO7* and *TYR1* act at differentiation steps leading to the production of certain amino acids over others, and *TRP5* is extensively involved in the formation of tryptophan. We presume heterozygous mutations in these genes cause decreased amino acid pools, altering TORC2 activity, and ultimately signaling for autophagy induction. In summary, we found that heterozygous mutations in aromatic amino acid biosynthesis genes can impact Tor signaling, supporting a connection between these pathways while also illuminating the sensitivity and preferentiality of TORC1 and TORC2 regulation.

## Methods

Yeast strains: Strain ES76 (Strome et al. 2008), genotype: MATa/MATα l *eu2-3/leu2-3, his3-Δ200/his3-Δ200, trp1-Δ1/trp1-Δ1, lys2-801/LYS2, ura3-52/ura3-52, can1-100/CAN1, ade2-101/ade2-101,* 2X [CF:( *ura3::TRP1, SUP11, CEN4, D8B* )] was used for creation of all strains used in this study.

Creation of heterozygous strains: Heterozygous deletions were achieved in the parental strain ES76 through insertion of a KanMX cassette in place of the gene of interest using long flanking homology based replacement (Wach et al. 1994). These KanMX cassettes containing large (200-800bp) regions of upstream and downstream flanking homology for each gene of interest were PCR amplified using KOD XL DNA Polymerase (Novagen #71087-3) from the *Saccharomyces cerevisiae* heterozygous deletion collection (Winzeler et al 1999) (Thermo Scientific YSC1055.) PCR-generated products were then transformed into parental strain ES76 using the lithium acetate method (Gietz and Woods 2006). Positive transformants were selected on 200μg/ml G418 plates and insertion site location was verified with an internal KanMX reverse primer and an upstream forward primer outside of the amplified cassette. Genotypes, primer information, and amplified PCR products for each created strain are described in full detail in Supplemental Table 1.

Quantitative real-time PCR (qRT-PCR) analysis of TORC1-dependent gene expression: RNA samples for strains of interest were prepared from isolated single colonies grown to log phase (0.4-0.9 OD600) in non-selective liquid YPD followed by TRIzol® reagent (Life Technologies #15596018) treatment using the standard provided protocol. Quantification of RNA was performed using a Nano-Drop NA-1000 spectrophotometer. Conversion of isolated RNA to cDNA was performed using the Verso cDNA Synthesis Kit (Thermo Scientific #AB-1453/B). Real-time PCR was performed using the DyNAmo Flash SYBR Green qPCR Kit DyNAmoColorFlash SYBR Green qPCR Kit (Thermo Scientific #F-416L) on an Applied Biosystems 7300 Real Time PCR System. The mRNA levels of TORC1-dependent genes *RPL9A, GAP1, MEP2,* as well as *TUB1* (internal control) were quantified using associated primer sets [Integrated DNA Technologies] (Hinnebusch 2005; Shen et al. 2007; Loewith and Hall 2011; Matsui et al. 2013) [See Supplemental Table 2]. Reactions were run as technical duplicates and collected Ct values were then used to calculate fold change for each strain/treatment of interest using the ΔΔCt method (Livak and Schmittgen 2001). Data was collected for a total of 6 biological replicate trials and fold changes for the performed trials were averaged. Positive controls were established by treatment of parental strains grown to log phase with either SD-N nitrogen starvation media (0.17% YNB without amino acids and ammonium sulfate, and 2% glucose (wt/vol) – filter sterilized) or amino acid starvation media (0.05% yeast extract, 2% wt/vol dextrose (Vlahakis and Powers 2014)) for 2 hours before RNA extraction, cDNA synthesis, and qRT-PCR analysis for comparison of expression to untreated parental strain (Matsui et al. 2013). Two-tailed t-tests with unequal variance were performed on ΔCt values to assess significance of qRT-PCR data. The Benjamini-Hochberg correction was used to account for multiple comparisons and statistical significance was determined based on a 5% false discovery rate.

Western blot analysis for changes in autophagy induction: Strains of interest were transformed using the lithium acetate method with the plasmid pRS316-GFP- *ATG8* kindly provided by Dr. Matsui and colleagues (Chiba University, Japan (Matsui et al. 2013)). The plasmid encodes a N-terminus GFP-fusion of the autophagy-related protein Atg8 (Matsui et al. 2013). Transformed strains were grown in SC-Ura for 18 hours before protein was extracted using the Yeast Protein Kit (Zymo Research #Y1002-1-6) with added protease inhibitor 4-(2-Aminoethyl)benzenesulfonyl fluoride hydrochloride (Thermo Scientific #78431). Positive controls were established by treatment of the wildtype ES76 strain transformed with the GFP- *ATG8* plasmid with either SD-N nitrogen starvation for 4 hours or amino acid starvation media for 4 hours after initial 18-hour growth in SC-Ura. Protein extracted from ES76 without the GFP- *ATG8* plasmid grown in YPD was included as a negative control. Isolated proteins were quantified through comparison to a standard curve generated using a bovine serum albumin standard (Life Technologies #23209) and CoomassiePlusTM Protein Assay Reagent (Thermo Scientific #1856210). Protein samples at a concentration of 160μg were treated with 2X SDS sample buffer, denatured at 95°C for 5 minutes, and cooled on ice for 2 minutes before being run in Mini-PROTEAN® TGXTM Precast Gels (Bio-Rad #456-9033) and transferred to PVDF membrane (Thermo Scientific #88518). Following transfer, the membrane was treated with blocking agent (5% skim milk in 1X TBST) for 1 hour before being treated with the primary Anti-GFP rabbit antibody (Abcam ab290) followed by the secondary Anti-Rabbit goat antibody HRP conjugate (Abcam ab97051). Treated membranes were scanned using WesternSureTM PREMIUM Chemiluminescent Substrate (LI-COR #926-95000) and a LI-COR C-Digit Scanner using the High Sensitivity setting and recommended background subtraction (Median Setting: Border Width 3, Right/Left Segment) with the associated ImageStudio Software. Bands were quantified using a standardized rectangle over bands of interest and compared to the same standardized rectangle placed adjacent to each band to quantify background signal (total in empty rectangle adjacent to band) to subtract from band signal (total in rectangle over band). After analysis of GFP-Atg8 and GFP expression, membranes were stripped and re-probed with primary Anti-Pgk1 mouse (Abcam ab22c5db) and secondary Anti-Mouse goat HRP conjugate (Invitrogen [G21040]) antibodies. Observed bands for GFP-Atg8 and GFP expression were then normalized to Pgk1p expression. Expression signals were averaged across four biological replicate trials used to calculate percent autophagy induction as follows: Normalized GFP signal/normalized GFP-Atg8 + normalized GFP signal. Percent induction values were then analyzed for significance using a two-tailed independent samples t-test of unequal variance to determine significance for changes in autophagy induction. This statistical test was chosen based on the need to analyze signal values from biological replicate samples across multiple trials while simultaneously accounting for high variance in signal value differences from trial to trial based on antibody reactivity. The Benjamini-Hochberg correction was used to account for multiple comparisons and statistical significance was determined based on a 5% false discovery rate.

## Extended Data

Description: Supplemental Table 1:&nbsp; Strain list and genotypes. Supplemental Table 2. qRT-PCR primer sets.. Resource Type: Text. DOI: 10.22002/xexp4-9ax68
